# Impact of thermal extrusion and microwave vacuum drying on fatty acids profile during fish powder preparation

**DOI:** 10.1002/fsn3.2236

**Published:** 2021-03-13

**Authors:** Nazir Ahmad, Abid Hussain, Sipper Khan, Sameh A. Korma, Ghulam Hussain, Rana Muhammad Aadil, Rabia Siddique, Anwar Ali, Umair Shabbir, Ahsan Ul Haq, Muhammad Faisal Manzoor, Azhari Siddeeg

**Affiliations:** ^1^ Institute of Food & Home Sciences Government College University Faisalabad Pakistan; ^2^ Department of Agriculture and Food Science Karakoram International University Gilgit Pakistan; ^3^ School of Food and Agriculture Sciences University of Management and Technology Lahore Pakistan; ^4^ Department of Food Science Faculty of Agriculture Zagazing University Sharkia Egypt; ^5^ School of Food Science and Engineering South China University of Technology Guangzhou China; ^6^ Department of Physiology Government College University Faisalabad Pakistan; ^7^ National Institute of Food Science and Technology University of Agriculture Faisalabad Pakistan; ^8^ Department of Chemistry Government College University Faisalabad Pakistan; ^9^ Department of Forestry and Range Management University of Agriculture Faisalabad Pakistan; ^10^ School of Food and Biological Engineering Jiangsu University Zhenjiang China; ^11^ Department of Food Engineering and Technology Faculty of Engineering and Technology University of Gezira Wad Medani Sudan

**Keywords:** extruded fish powder, fatty acids, peroxide value, vacuum drying

## Abstract

The current study aimed to optimize the process for ready‐to‐eat extruded fish powder preparation and to ascertain the impact of two methods on fatty acid profiles. For the investigation, *Labeo rohita* (Rohu) fish was first minced, extruded, and microwave vacuum‐dried. The results show that the yield for extruded fish powder (EFP) fluctuated from 22.32% to 29.07%. The maximum retention for docosahexaenoic acid (DHA), arachidonic acid (AA), and eicosapentaenoic acid (EPA) was 3.24 ± 0.08 g/100 g lipids, 2.74 ± 0.05 g/100 g lipids, and 1.24 ± 0.09 g/100 g lipids, by using different extrusion parameters. Moreover, nonsignificant changes were observed during 0 days, and 1 and 3 months of storage (at 4°C and 25°C) for DHA, AA, and EPA, whereas significant results were recorded for the samples stored for 6 months at 25°C. Also, the maximum peroxide value (PV) and thiobarbituric acid reactive substance values (TBARS) were 1.72 ± 0.04 meq/kg fat and 0.135 ± 0.008 mg malondialdehyde/kg fat. It is anticipated from the outcomes that the study will be helpful to prepare value‐added food products in future studies.

## Graphical abstract

Nowadays, the dried fish powder (FP) can be applied for the production of value‐added foods and food fortification has supported the food industry to investigate various approaches for producing FP from multiple raw materials. The aim of this study was to optimize the process of ready‐to‐eat extruded fish powder preparation and its characterization for bioactive fatty acids. Our results report that the processing methods such as thermal extrusion and microwave vacuum drying improve the bioactive fatty acids, PV, and TBARS of FP which is very useful for the food industry to manufacture value‐added functional products.

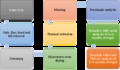

## 1 INTRODUCTION

Fish is among the most common and nutritious seafood consumed throughout the globe. In Pakistan, the total fish production in the year 2017–2018 was estimated as 482,000 million tons (Govt. of Pakistan, 2017). Among different fish varieties, Rohu is mostly consumed in Pakistan and India (Latif & Faheem, 2020), due to its nutritional and sensorial attributes. Numerous studies have proved the bioactive composition of fish meat which helps in preventing cardiovascular ailments, atherosclerosis, acts as antithrombotic, and functions against arrhythmia (Šimat et al., 2020; Ucak et al., 2021).

However, being perishable and high‐fat content, the seafood is enlisted in a highly perishable food class. Therefore, various processing technologies are being used to develop fish products for long‐term consumption and storage. In addition to other sea processed products, fish powder (FP) consumption is increasing and it acts as an important ingredient for enriched foodstuff (Manzoor et al., 2021; Shaviklo et al., 2010). FP is reported to carry polyunsaturated fatty acids (PUFAs) including docosahexaenoic acid (DHA), arachidonic acid (AA), and eicosapentaenoic acid (EPA). The health‐promoting fatty acid profile acts as antithrombotic and hypocholesterolemic, and performs other physiological actions (Tocher, 2015). Furthermore, DHA is helpful for brain development (Echeverría et al., 2017) and eye retina of newborn children (Jacques et al., 2011). Similarly, AA acts as a precursor in products such as anandamide, isoprostanes, and epoxides (Tallima & El Ridi, 2018). The National Institute of the United States recommended 650 mg of fish fatty acids daily (Chew et al., 2015).

However, PUFAs are highly susceptible to oxidative which can render the quality of fish and make it unfit for consumption. Novel processing approaches helped to prepare FP, having a longer shelf life. Extrusion is one of the processing technologies in which high temperature and smear force are used to produce a product with improved biochemical characteristics (Ahmad et al., 2020). This study aimed to process Rohu fish into dried extruded fish powder (EFP), with limited oxidation of bioactive fatty acids.

## 2 MATERIALS AND METHODS

### 2.1 Procurement and preparation of fish meat

Rohu fish (1,400 ± 150 g) was purchased from the local market of Faisalabad, Pakistan. Fish guts (fins, head, and tail) were removed following deboning and mincing. A spinning blade grinder (Duronic CG250‐250W motor, UK) was used for mincing, then exposed to thermal extrusion, and finally, microwave vacuum‐dried to form FP as the final product.

### 2.2 Thermal extrusion

The twin‐screw extruder was used for thermal extrusion (FMHE‐36; Hunan Fumake Engineering Technology Co., Ltd., Hunan, China). It had a screw width of 36 mm with a length to distance proportion of 24:1 and kick the bucket measurement of 36 × 4 mm. It had controlled zones for thermal cooking with a temperature estimating test and a screw speed controller. The Box–Behnken design was used for the optimization. The conditions of barrel external temperature (BET) for the optimization ranging from 100 to 150°C, screw speed (SS) of 50–150 rpm, feed flow rate (FFR) of 30–90 kg/hr, feed moisture content (FMC) of 10%–30%, and FP 0%–25%. Coding techniques were used for the experimental design shown in Table 1.

**TABLE 1:** Codes and actual levels of independent variables for optimization of response factors determined by the Box–Behnken design

Independent variables	Units	Coded levels		
		−1	0	+1
Barrel exit temperature (BET)	°C	100	125	150
Feed flow rate (FFR)	kg/hr	30	60	90
Screw speed (SS)	rpm	50	100	150
Feed moisture content (FMC)	%	10	20	30

### 2.3 Microwave vacuum drying

For the drying of samples, a small‐scale vacuum dryer (WZD2S; Nanjing Sanle, China) was employed. The equipment had the potential to dry 8–12 kg fish samples in controlled temperature and pressure. The dryer was adjusted to 1,300 W power, the frequency was set to 2,450 MHz frequency, and a sample was kept at 90 Pascal pressure for 4.5 hr (50°C). For keeping the quality of n‐3 and n‐6 fatty acids in the finished product, low temperature and pressure were used in the study.

### 2.4 Grinding and yield

Grinding was done to produce a homogenous particle size of powder using a grinder (Duronic CG250‐250W motor, UK). The dried powder was successfully packed in polyethylene bags. The yield was calculated according to the AOAC method (923.07) (Latimer, 2012).

### 2.5 Proximate analyses

Moisture content was analyzed using AACC Method No. 44‐15A (AACC, 2000). Protein content was determined by using AACC Method No. 46‐10 (AACC, 2000). By Soxtec System (Model: H‐2 1045 Extraction Unit, Hoganas, Sweden), fat was determined AACC Method No. 30‐10 (AACC, 2000). By using AACC Method No. 32‐10, carbohydrates (CHO) were measured (AACC, 2000). Using Muffle Furnace (MF‐1/02, PCSIR, Pakistan), mineral contents were determined according to AACC Method No. 08‐01 (AACC, 2000).

### 2.6 Preparation of sample for fatty acid (FA) analysis

PUFAs of EFP were calculated according to AOAC, Method No. 923.07 (Latimer, 2012). Fatty acid methyl esters (FAME) were synthesized by following the method proposed by Carvalho et al. (2005). In the tube (16 × 150 mm), 1 g sample was taken in tube (16 × 150 mm) and then added 10 ml of hexane containing 0.1% butylated hydroxyl toluene. After that mixed by shaking and placed in an ultrasound water bath for 5 min. It was centrifuged at 1,500 *g*. The hexane was heated at 60°C to evaporate it from the sample mixture. Aspirated sample with nitrogen for 10 s, in 50 mg of sample, 1.0 ml toluene, and 2.0 ml boron trichloride–methanol. The samples were heated further at 60°C for 10 min. Finally, 2.0 ml of hexane and 2.0 ml of cooled water were added to a test tube for the extraction of FAME. The moisture was removed by adding anhydrous sodium sulfate, and dehydrated FAME was transferred to a volumetric flask (10 ml) and made up the volume with hexane.

### 2.7 Gas chromatographic assessment

For GC analysis, 1.0 μl FAME sample was introduced with helium (as carrier gas) with a speed of 1 ml/min, whereas the column oven temperature was adjusted to 160°C with a gradual increase in 3°C/min until it reaches 180°C. Additionally, the column oven temperature was raised from 180°C to 220°C at 1°C/min speed. At 220°C, it was kept for 7.5 min; furthermore, the split ratio was set at 50% with injector temperature of 240°C and detector temperature of 250°C.

### 2.8 Retention of FAs

For FA retention calculations, the following equation was employed:

$$\text{FAs retention}\;\left(\%\right)=\frac{\text{FAs present in the product after extrusion}}{\text{FAs present in raw material}}\times 100$$

### 2.9 Peroxide value analysis

A sample of 5 g FP was taken in a conical flask. 30 ml of acetic acid, 20 ml chloroform, and 1 ml potassium iodide solution were added to it and stayed in a dark place for 30 min. 1 ml of starch solution (1%) and 50 ml of water were mixed into the solution. The final solution was then titrated with sodium thiosulfate solution (0.1 N) until the colorless endpoint is obtained (Ranganna, 1986). The following equation was used to determine the PV of FP.

$$\text{PV}=\frac{\text{Titrate value}\times {\text{Normality of Na}}_{2}S_{2}O_{3}}{\text{Sample weight}}\times 1000$$

### 2.10 Thiobarbituric acid reactive substance (TBARS) test

For TBARS analysis, 5 g of sample was first homogenized with trichloroacetic acid (11%) for 1 min (at 5,400 *g*) using a homogenizer (IKA, Wilmington, USA). Then, the sample was kept in ice for 1 min followed by homogenization for 1 min again. Further, separation of homogenate was performed with Whatman's No. 1 and mixed 1 ml (20 mM) thiobarbituric acid followed by incubation for 20 hr (at 25°C) in a darkroom. Finally, the absorbance was ascertained using a UV‐1800 spectrophotometer (Shimadzu, Kyoto, Japan) at 532 nm wavelength. The obtained results were calculated in mg of MDA/kg.

### 2.11 Statistical analysis

The optimization of microwave and extrusion conditions was performed through RSM using Design‐Expert 11 software of statistics. The experiment analyses were conducted through the method of Montgomery (1991). All experiments were conducted in triplicates and average values were considered as mean values. The significance of values was calculated statistically through mean using analysis of variance (ANOVA) at a probability of 0.05.

## 3 RESULTS

### 3.1 Yield and proximate composition of fish and EFP

The effects of thermal extrusion conditions of BET, SS, FFR, and FMC were examined for EFP preparation, yield, and retention of DHA, EPA, and AA. The average yield for EFP as a result of different operating conditions differed from 22.32% to 29.07%. Whereas the total fat content was 9.62 ± 0.43% and the total crude protein was 67.28 ± 1.09% in EFP, the values for CHO, moisture, and ash were 11.55 ± 0.78%, 4.52 ± 0.31%, and 6.93 ± 0.90%, respectively (Table 2). The fatty acid profile of fish meat and EFP is presented in Table 3. The values for PUFAs, monounsaturated fatty acids (MUFAs), saturated fatty acids (SFAs), n‐3, and n‐6 in fresh fish were 51.73 ± 2.87, 29.58 ± 1.43, 20.06 ± 1.7, 9.26 ± 0.99, and 10.39 ± 0.68, while for EFP, the values were reduced to 50.00 ± 0.64, 27.71 ± 1.26, 17.98 ± 0.72, 8.28 ± 0.41, and 9.33 ± 0.28 (Table 3).

**TABLE 2:** Proximate composition of Rohu fish and its powder prepared by microwave vacuum drying

*Labeo rohita* (Rohu)	Total fat (%)	Total protein (%)	Moisture (%)	Ash (%)
Control	2.74 ± 0.13	14.94 ± 0.72	75.46 ± 1.71	2.41 ± 0.37
EFP	9.62 ± 0.43	67.28 ± 1.09	4.52 ± 0.31	8.93 ± 0.90

**TABLE 3:** Fatty acid profile in fresh Rohu fish and in fish powder (mg/100 g)

Fatty acid	Control	EFP
C14:0	2.47 ± 0.21	2.37 ± 0.05
C15:0	1.10 ± 0.07	1.04 ± 0.03
C16:0	33.09 ± 1.68	31.82 ± 0.31
C17:0	1.30 ± 0.04	1.27 ± 0.01
C18:0	13.12 ± 0.8	12.59 ± 0.15
C19:0	0.65 ± 0.05	0.62 ± 0.02
C20:0	0.29 ± 0.02	0.29 ± 0.07
C16:1 n‐7	0.70 ± 0.04	0.62 ± 0.09
C18:1 n‐9	26.91 ± 1.30	25.29 ± 1.13
C20:1 n‐9	1.97 ± 0.09	1.80 ± 0.04
C18: 2 n‐6	4.53 ± 0.52	3.98 ± 0.13
C18: 3 n‐3	2.91 ± 0.19	2.62 ± 0.19
C20:3 n‐7	0.41 ± 0.03	0.37 ± 0.03
C20:5 n‐3 (EPA)	1.32 ± 0.06	1.24 ± 0.03
C20:4 n‐6 (AA)	2.91 ± 0.12	2.74 ± 0.14
C24:4 n‐6	2.95 ± 0.16	2.61 ± 0.15
C25:5 n‐3	1.34 ± 0.37	1.18 ± 0.03
C22:6 n‐3 (DHA)	3.69 ± 0.43	3.24 ± 0.19
∑SFA	51.73 ± 2.87	50.00 ± 0.64
∑MUFA	29.58 ± 1.43	27.71 ± 1.26
∑PUFA	20.06 ± 1.7	17.98 ± 0.72
∑ n3	9.26 ± 0.99	8.28 ± 0.41
∑ n6	10.39 ± 0.68	9.33 ± 0.28
n3/n6	0.89	0.88

### 3.2 Retention of DHA, EPA, and AA

The trend of various conditions on DHA, EPA, and AA values is presented in Tables 4, 5, 6. An increasing trend of DHA, EPA, and AA retention was observed with decreasing temperature and increasing FMC. The minimum retention value for DHA was 2.34 ± 0.04 g/100 g lipid at 150 BET, 100 SS, 60 FFR, and 10 FMC, while the maximum value was observed 3.24 ± 0.08 g/100 g lipids at 100°C BET, 100 SS, 60 FFR, and at 30 FMC (Table 4). Similarly, the minimum value observed for EPA at 150 BET, 100 SS, 60 FFR, and 10 FMC was 0.33 ± 0.003 g/100 g lipid and the maximum result was 1.24 ± 0.09 g/100 g lipid at 100 BET, 100 SS, 60 FFR, and 30 FMC as displayed in Table 5. The lowest values estimated for AA were 1.81 ± 0.06 g/100 g lipid at 150 BET, 100 SS, 60 FFR, and 10 FMC, while the highest estimation was 2.74 ± 0.05 g/100 g lipids at 100 BET, 100 SS, 60 FFR, and 30 FMC.

**TABLE 4:** Retention and storage stability of DHA in EFP at different temperature and storage intervals

Extrusion processing run	Independent variables				Dependent variable						
	Barrel exit temp. (BET) °C	Screw speed (SS)	Feed flow rate (FFR)	Feed moisture content (FMC)	DHA (gram/100 g lipid)						
					0 day	Storage at 4°C			Storage at 25°C		
						1 month	3 months	6 months	1 month	3 months	6 months
1	150 (1)	150 (1)	60 (0)	20 (0)	2.48 ± 0.01^mn^	2.48 ± 0.03^mn^	2.48 ± 0.02^mn^	2.45 ± 0.03^n^	2.48 ± 0.01^mn^	2.47 ± 0.03^mn^	2.45 ± 0.01^n^
2 (C1)	125 (0)	100 (0)	60 (0)	20 (0)	2.70 ± 0.03^j^	2.70 ± 0.02^j^	2.70 ± 0.06^j^	2.69 ± 0.05^j^	2.70 ± 0.02^j^	2.69 ± 0.01^j^	2.67 ± 0.02^j^
3	125 (0)	150 (1)	60 (0)	10 (−1)	2.64 ± 0.01^k^	2.64 ± 0.05^k^	2.64 ± 0.03^k^	2.64 ± 0.02^k^	2.64 ± 0.05^k^	2.64 ± 0.03^k^	2.62 ± 0.05^k^
4	125 (0)	100 (0)	30 (−1)	10 (−1)	2.64 ± 0.05^k^	2.64 ± 0.03^k^	2.64 ± 0.04^k^	2.64 ± 0.03^k^	2.64 ± 0.01^k^	2.64 ± 0.02^k^	2.62 ± 0.01^k^
5	125 (0)	100 (0)	90 (1)	30 (1)	2.87 ± 0.02^g^	2.87 ± 0.05^g^	2.87 ± 0.03^g^	2.86 ± 0.01^g^	2.87 ± 0.03^g^	2.85 ± 0.04^g^	2.80 ± 0.03^h^
6	150 (1)	100 (0)	60 (0)	10 (−1)	2.34 ± 0.04^p^	2.34 ± 0.08^p^	2.34 ± 0.05^p^	2.33 ± 0.02^p^	2.34 ± 0.01^p^	2.33 ± 0.02^p^	2.30 ± 0.02^pq^
7	125 (0)	100 (0)	90 (1)	10 (−1)	2.63 ± 0.03^k^	2.63 ± 0.02^k^	2.63 ± 0.01^k^	2.63 ± 0.04^k^	2.63 ± 0.05^k^	2.63 ± 0.04^k^	2.61 ± 0.05^kl^
8 (C2)	125 (0)	100 (0)	60 (0)	20 (0)	2.71 ± 0.01^ij^	2.71 ± 0.03^ij^	2.71 ± 0.02^ij^	2.71 ± 0.01^ij^	2.71 ± 0.02^ij^	2.71 ± 0.01^ij^	2.69 ± 0.02^j^
9	125 (0)	50 (−1)	90 (1)	20 (0)	2.73 ± 0.02^ij^	2.73 ± 0.05^ij^	2.73 ± 0.06^ij^	2.72 ± 0.05^ij^	2.73 ± 0.03^ij^	2.72 ± 0.02^ij^	2.70 ± 0.04^j^
10	125 (0)	50 (−1)	30 (−1)	20 (0)	2.74 ± 0.05^ij^	2.74 ± 0.04^ij^	2.74 ± 0.02^ij^	2.73 ± 0.03^ij^	2.74 ± 0.06^ij^	2.73 ± 0.05^ij^	2.71 ± 0.07^ij^
11	100 (−1)	100 (0)	90 (1)	20 (0)	3.15 ± 0.03^bc^	3.15 ± 0.07^bc^	3.16 ± 0.08^b^	3.15 ± 0.06^bc^	3.15 ± 0.05^bc^	3.12 ± 0.06^bc^	3.05 ± 0.06^de^
12	125 (0)	150 (1)	30 (−1)	20 (0)	2.73 ± 0.02^ij^	2.73 ± 0.03^ij^	2.73 ± 0.01^ij^	2.73 ± 0.04^ij^	2.73 ± 0.02^ij^	2.72 ± 0.04^ij^	2.70 ± 0.03^j^
13	100 (−1)	100 (0)	30 (−1)	20 (0)	3.13 ± 0.04^bc^	3.13 ± 0.01^bc^	3.13 ± 0.05^bc^	3.12 ± 0.02^bc^	3.12 ± 0.01^bc^	3.10 ± 0.02^cd^	3.04 ± 0.05^de^
14	150 (1)	50 (−1)	60 (0)	20 (0)	2.43 ± 0.06^no^	2.43 ± 0.05^no^	2.43 ± 0.03^no^	2.43 ± 0.04^no^	2.43 ± 0.06^no^	2.43 ± 0.07^no^	2.41 ± 0.08^no^
15	150 (1)	100 (0)	90 (1)	20 (0)	2.45 ± 0.01^n^	2.45 ± 0.03^n^	2.45 ± 0.02^n^	2.49 ± 0.01^mn^	2.44 ± 0.02^n^	2.43 ± 0.01^no^	2.47 ± 0.01^mn^
16	100 (−1)	100 (0)	60 (0)	30 (1)	3.24 ± 0.08^a^	3.24 ± 0.06^a^	3.24 ± 0.04^a^	3.21 ± 0.03^a^	3.24 ± 0.01^a^	3.23 ± 0.04^a^	3.10 ± 0.02^cd^
17 (C3)	125 (0)	100 (0)	60 (0)	20 (0)	2.70 ± 0.02^j^	2.70 ± 0.04^j^	2.70 ± 0.01^j^	2.69 ± 0.02^j^	2.70 ± 0.03^j^	2.69 ± 0.02^j^	2.66 ± 0.03^jk^
18	150 (1)	100 (0)	30 (−1)	20 (0)	2.42 ± 0.04^no^	2.42 ± 0.02^no^	2.42 ± 0.03^no^	2.41 ± 0.04^no^	2.42 ± 0.02^no^	2.41 ± 0.03^no^	2.39 ± 0.01^o^
19	100 (−1)	100 (0)	60 (0)	10 (−1)	3.06 ± 0.08^d^	3.06 ± 0.07^d^	3.06 ± 0.09^d^	3.04 ± 0.08^de^	3.04 ± 0.07^de^	3.01 ± 0.06^de^	2.98 ± 0.05^ef^
20	150 (1)	100 (0)	60 (0)	30 (1)	2.56 ± 0.01^l^	2.56 ± 0.03^l^	2.56 ± 0.02^l^	2.56 ± 0.01^l^	2.55 ± 0.03^lm^	2.55 ± 0.01^lm^	2.51 ± 0.02^m^
21(C4)	125 (0)	100 (0)	60 (0)	20 (0)	2.71 ± 0.03^ij^	2.70 ± 0.02^j^	2.70 ± 0.01^j^	2.70 ± 0.04^j^	2.70 ± 0.05^j^	2.69 ± 0.04^j^	2.67 ± 0.05^j^
22	125 (0)	50 (−1)	60 (0)	10 (−1)	2.62 ± 0.06^k^	2.62 ± 0.05^k^	2.62 ± 0.04^k^	2.61 ± 0.02^kl^	2.62 ± 0.01^k^	2.61 ± 0.02^kl^	2.57 ± 0.01^l^
23 (C5)	125 (0)	100 (0)	60 (0)	20 (0)	2.71 ± 0.02^ij^	2.71 ± 0.04^ij^	2.71 ± 0.03^ij^	2.70 ± 0.01^j^	2.71 ± 0.04^ij^	2.70 ± 0.03^j^	2.65 ± 0.02^k^
24	100 (−1)	150 (1)	60 (0)	20 (0)	3.14 ± 0.08^bc^	3.14 ± 0.07^bc^	3.14 ± 0.06^bc^	3.14 ± 0.09^bc^	3.14 ± 0.08^bc^	3.11 ± 0.07^c^	3.07±0.0.08^cd^
25	125 (0)	50 (−1)	60 (0)	30 (1)	2.84 ± 0.04^gh^	2.84 ± 0.06^gh^	2.84 ± 0.03^gh^	2.83 ± 0.05^gh^	2.84 ± 0.04^gh^	2.83 ± 0.02^gh^	2.77 ± 0.03^hi^
26	125 (0)	100 (0)	30 (−1)	30 (1)	2.85 ± 0.03^g^	2.85 ± 0.05^g^	2.85 ± 0.07^g^	2.84 ± 0.06^gh^	2.85 ± 0.08^g^	2.84 ± 0.09^gh^	2.78 ± 0.07^hi^
27	125 (0)	150 (1)	90 (1)	20 (0)	2.75 ± 0.08^i^	2.75 ± 0.09^i^	2.75 ± 0.05^i^	2.75 ± 0.04^i^	2.75 ± 0.03^i^	2.74 ± 0.04^ij^	2.72 ± 0.02^ij^
28	125 (0)	150 (1)	60 (0)	30 (1)	2.86 ± 0.02^g^	2.86 ± 0.04^g^	2.86 ± 0.02^g^	2.85 ± 0.01^g^	2.86 ± 0.02^g^	2.85 ± 0.01^g^	2.78 ± 0.03^hi^
29	100 (−1)	50 (−1)	60 (0)	20 (0)	2.95 ± 0.05^f^	2.95 ± 0.07^f^	2.95 ± 0.04^f^	2.94 ± 0.03^f^	2.94 ± 0.04^f^	2.91 ± 0.03^fg^	2.76 ± 0.01^hi^

Note

C1, C2, C3, C4, and C5 are the center points where extrusion processing was applied. Different superscripts letters indicate the significance difference (*p* ≤ .05) within the rows and columns within DHA.

Abbreviations: BET, barrel exit temperature; DHA, docosahexaenoic acid; FFR, feed flow rate; FMC, feed moisture content; FP, fish powder; SS, screw speed.

**TABLE 5:** Retention and storage stability of EPA in EFP at different temperature and storage intervals

Extrusion processing run	Independent variables				Dependent variable						
	Barrel exit temp. (BET) °C	Screw speed (SS)	Feed flow rate (FFR)	Feed moisture content (FMC)	EPA (gram/100 g lipid)						
					0 day	Storage at 4°C			Storage at 25°C		
						1 month	3 months	6 months	1 month	3 months	6 months
1	150 (1)	150 (1)	60 (0)	20 (0)	0.46 ± 0.004^m^	0.46 ± 0.001^m^	0.46 ± 0.002^m^	0.45 ± 0.007^mn^	0.46 ± 0.005^m^	0.45 ± 0.003^mn^	0.43 ± 0.002^mn^
2 (C1)	125 (0)	100 (0)	60 (0)	20 (0)	0.81 ± 0.006^h^	0.81 ± 0.002^h^	0.81 ± 0.005^h^	0.81 ± 0.001^h^	0.81 ± 0.007^h^	0.80 ± 0.005^hi^	0.77 ± 0.001^hi^
3	125 (0)	150 (1)	60 (0)	10 (−1)	0.70 ± 0.001^j^	0.70 ± 0.008^j^	0.70 ± 0.002^j^	0.70 ± 0.005^j^	0.70 ± 0.008^j^	0.69 ± 0.003^j^	0.67 ± 0.006^jk^
4	125 (0)	100 (0)	30 (−1)	10 (−1)	0.69 ± 0.004^j^	0.69 ± 0.001^j^	0.69 ± 0.005^j^	0.69 ± 0.006^j^	0.69 ± 0.003^j^	0.68 ± 0.001^jk^	0.66 ± 0.009^jk^
5	125 (0)	100 (0)	90 (1)	30 (1)	0.93 ± 0.007^f^	0.93 ± 0.008^f^	0.92 ± 0.001^f^	0.91 ± 0.007^fg^	0.92 ± 0.004^f^	0.89 ± 0.004^fg^	0.81 ± 0.007^h^
6	150 (1)	100 (0)	60 (0)	10 (−1)	0.33 ± 0.003^o^	0.33 ± 0.002^o^	0.33 ± 0.005^o^	0.33 ± 0.008^o^	0.33 ± 0.004^o^	0.31 ± 0.002^op^	0.29 ± 0.006^op^
7	125 (0)	100 (0)	90 (1)	10 (−1)	0.70 ± 0.005^j^	0.70 ± 0.006^j^	0.70 ± 0.007^j^	0.70 ± 0.002^j^	0.70 ± 0.006^j^	0.69 ± 0.006^j^	0.67 ± 0.001^jk^
8 (C2)	125 (0)	100 (0)	60 (0)	20 (0)	0.81 ± 0.006^h^	0.81 ± 0.005^h^	0.81 ± 0.001^h^	0.80 ± 0.004^hi^	0.81 ± 0.002^h^	0.80 ± 0.003^hi^	0.77 ± 0.005^hi^
9	125 (0)	50 (−1)	90 (1)	20 (0)	0.79 ± 0.002^hi^	0.79 ± 0.001^hi^	0.79 ± 0.003^hi^	0.78 ± 0.007^hi^	0.79 ± 0.005^hi^	0.78 ± 0.004^hi^	0.76 ± 0.001^i^
10	125 (0)	50 (−1)	30 (−1)	20 (0)	0.78 ± 0.001^hi^	0.78 ± 0.008^hi^	0.78 ± 0.002^hi^	0.76 ± 0.006^i^	0.78 ± 0.001^hi^	0.77 ± 0.006^hi^	0.75 ± 0.004^i^
11	100 (−1)	100 (0)	90 (1)	20 (0)	1.14 ± 0.07^bc^	1.14 ± 0.03^bc^	1.14 ± 0.05^bc^	1.12 ± 0.06^bc^	1.14 ± 0.08^bc^	1.11 ± 0.04^c^	1.06 ± 0.01^d^
12	125 (0)	150 (1)	30 (−1)	20 (0)	0.83 ± 0.008^gh^	0.83 ± 0.001^gh^	0.83 ± 0.004^gh^	0.83 ± 0.005^gh^	0.83 ± 0.007^gh^	0.83 ± 0.005^gh^	0.80 ± 0.006^hi^
13	100 (−1)	100 (0)	30 (−1)	20 (0)	1.13 ± 0.05^bc^	1.13 ± 0.07^bc^	1.13 ± 0.01^bc^	1.13 ± 0.02^bc^	1.13 ± 0.04^bc^	1.11 ± 0.06^c^	1.05 ± 0.05^de^
14	150 (1)	50 (−1)	60 (0)	20 (0)	0.44 ± 0.001^mn^	0.44 ± 0.003^mn^	0.44 ± 0.005^mn^	0.44 ± 0.008^mn^	0.44 ± 0.008^mn^	0.44 ± 0.005^mn^	0.43 ± 0.001^mn^
15	150 (1)	100 (0)	90 (1)	20 (0)	0.45 ± 0.003^mn^	0.45 ± 0.005^mn^	0.45 ± 0.009^mn^	0.45 ± 0.001^mn^	0.45 ± 0.003^mn^	0.43 ± 0.006^mn^	0.41 ± 0.007^n^
16	100 (−1)	100 (0)	60 (0)	30 (1)	1.24 ± 0.09^a^	1.24 ± 0.05^a^	1.24 ± 0.08^a^	1.24 ± 0.06^a^	1.24 ± 0.04^a^	1.22 ± 0.07^a^	1.15 ± 0.02^bc^
17 (C3)	125 (0)	100 (0)	60 (0)	20 (0)	0.80 ± 0.003^hi^	0.80 ± 0.005^hi^	0.80 ± 0.006^hi^	0.80 ± 0.002^hi^	0.80 ± 0.003^hi^	0.79 ± 0.005^hi^	0.78 ± 0.008^hi^
18	150 (1)	100 (0)	30 (−1)	20 (0)	0.86 ± 0.005^g^	0.86 ± 0.003^g^	0.86 ± 0.001^g^	0.85 ± 0.006^gh^	0.86 ± 0.004^g^	0.85 ± 0.001^gh^	0.83 ± 0.004^gh^
19	100 (−1)	100 (0)	60 (0)	10 (−1)	1.04 ± 0.02^de^	1.04 ± 0.01^de^	1.04 ± 0.06^de^	1.04 ± 0.08^de^	1.03 ± 0.05^de^	1.02 ± 0.03^de^	0.98 ± 0.009^e^
20	150 (1)	100 (0)	60 (0)	30 (1)	0.53 ± 0.004^L^	0.53 ± 0.002^L^	0.53 ± 0.003^L^	0.52 ± 0.001^L^	0.53 ± 0.003^L^	0.52 ± 0.005^L^	0.50 ± 0.008^Lm^
21(C4)	125 (0)	100 (0)	60 (0)	20 (0)	0.81 ± 0.009^h^	0.81 ± 0.006^h^	0.81 ± 0.008^h^	0.81 ± 0.007^h^	0.81 ± 0.005^h^	0.80 ± 0.008^hi^	0.78 ± 0.006^hi^
22	125 (0)	50 (−1)	60 (0)	10 (−1)	0.67 ± 0.009^jk^	0.67 ± 0.005^jk^	0.67 ± 0.007^jk^	0.66 ± 0.008^jk^	0.67 ± 0.006^jk^	0.64 ± 0.004^k^	0.63 ± 0.007^k^
23 (C5)	125 (0)	100 (0)	60 (0)	20 (0)	0.80 ± 0.003^hi^	0.80 ± 0.005^hi^	0.80 ± 0.004^hi^	0.79 ± 0.002^hi^	0.80 ± 0.007^hi^	0.77 ± 0.005^hi^	0.76 ± 0.009^i^
24	100 (−1)	150 (1)	60 (0)	20 (0)	1.16 ± 0.02^b^	1.16 ± 0.03^b^	1.16 ± 0.07^b^	1.16 ± 0.04^b^	1.16 ± 0.07^b^	1.14 ± 0.05^bc^	1.07 ± 0.08^cd^
25	125 (0)	50 (−1)	60 (0)	30 (1)	0.91 ± 0.001^fg^	0.91 ± 0.004^fg^	0.90 ± 0.006^fg^	0.90 ± 0.003^fg^	0.91 ± 0.007^fg^	0.87 ± 0.003^g^	0.84 ± 0.002^gh^
26	125 (0)	100 (0)	30 (−1)	30 (1)	0.92 ± 0.003^f^	0.92 ± 0.005^f^	0.93 ± 0.006^f^	0.93 ± 0.008^f^	0.92 ± 0.004^f^	0.89 ± 0.002^fg^	0.85 ± 0.005^gh^
27	125 (0)	150 (1)	90 (1)	20 (0)	0.84 ± 0.002^gh^	0.84 ± 0.005^gh^	0.84 ± 0.003^gh^	0.84 ± 0.005^gh^	0.84 ± 0.002^gh^	0.83 ± 0.006^gh^	0.80 ± 0.004^hi^
28	125 (0)	150 (1)	60 (0)	30 (1)	0.95 ± 0.007^ef^	0.95 ± 0.002^ef^	0.96 ± 0.006^ef^	0.96 ± 0.003^ef^	0.95 ± 0.005^ef^	0.94 ± 0.007^ef^	0.89 ± 0.003^fg^
29	100 (−1)	50 (−1)	60 (0)	20 (0)	1.12 ± 0.05^bc^	1.12 ± 0.08^bc^	1.12 ± 0.04^bc^	1.12 ± 0.09^bc^	1.12 ± 0.07^bc^	1.10 ± 0.03^cd^	1.04 ± 0.05^de^

Note

C1, C2, C3, C4, and C5 are the center points where extrusion processing was applied. Different superscripts letters indicate the significance difference (*p* ≤ .05) within the rows and columns within DHA.

Abbreviations: BET, barrel exit temperature; EPA, eicosapentaenoic acid; FFR, feed flow rate; FMC, feed moisture content; FP, fish powder; SS, screw speed.

**TABLE 6:** Retention and storage stability of AA in EFP at different temperature and storage intervals

Extrusion processing run	Independent variables				Dependent variable						
	Barrel exit temp. (BET) °C	Screw speed (SS)	Feed flow rate (FFR)	Feed moisture content (FMC)	AA (gram/100 g lipid)						
					0 day	Storage as 4°C			Storage as 25°C		
						1 month	3 months	6 months	1 month	3 months	6 months
1	150 (1)	150 (1)	60 (0)	20 (0)	1.91 ± 0.01^no^	1.91 ± 0.03^no^	1.91 ± 0.05^no^	1.90 ± 0.03^no^	1.91 ± 0.08^no^	1.90 ± 0.05^no^	1.88 ± 0.03^o^
2 (C1)	125 (0)	100 (0)	60 (0)	20 (0)	2.26 ± 0.03^ij^	2.26 ± 0.05^ij^	2.26 ± 0.04^ij^	2.26 ± 0.07^ij^	2.26 ± 0.09^ij^	2.25 ± 0.04^ij^	2.22 ± 0.06^j^
3	125 (0)	150 (1)	60 (0)	10 (−1)	2.17 ± 0.05^k^	2.17 ± 0.08^k^	2.17 ± 0.01^k^	2.17 ± 0.06^k^	2.17 ± 0.05^k^	2.17 ± 0.08^k^	2.15 ± 0.09^kl^
4	125 (0)	100 (0)	30 (−1)	10 (−1)	2.16 ± 0.07^k^	2.16 ± 0.05^k^	2.16 ± 0.07^k^	2.16 ± 0.09^k^	2.16 ± 0.04^k^	2.16 ± 0.03^k^	2.14 ± 0.07^kl^
5	125 (0)	100 (0)	90 (1)	30 (1)	2.37 ± 0.08^g^	2.37 ± 0.04^g^	2.38 ± 0.02^g^	2.37 ± 0.06^g^	2.37 ± 0.07^g^	2.35 ± 0.04^gh^	2.32 ± 0.01^h^
6	150 (1)	100 (0)	60 (0)	10 (−1)	1.81 ± 0.06^p^	1.81 ± 0.05^p^	1.81 ± 0.03^p^	1.81 ± 0.07^p^	1.81 ± 0.03^p^	1.81 ± 0.01^p^	1.80 ± 0.09^p^
7	125 (0)	100 (0)	90 (1)	10 (−1)	2.16 ± 0.07^k^	2.16 ± 0.03^k^	2.16 ± 0.01^k^	2.16 ± 0.08^k^	2.16 ± 0.04^k^	2.16 ± 0.02^k^	2.14 ± 0.07^kl^
8 (C2)	125 (0)	100 (0)	60 (0)	20 (0)	2.27 ± 0.08^i^	2.27 ± 0.03^i^	2.27 ± 0.07^i^	2.27 ± 0.05^i^	2.27 ± 0.08^i^	2.27 ± 0.09^i^	2.25 ± 0.01^ij^
9	125 (0)	50 (−1)	90 (1)	20 (0)	2.26 ± 0.04^ij^	2.26 ± 0.01^ij^	2.26 ± 0.04^ij^	2.26 ± 0.02^ij^	2.26 ± 0.06^ij^	2.26 ± 0.04^ij^	2.26 ± 0.03^ij^
10	125 (0)	50 (−1)	30 (−1)	20 (0)	2.25 ± 0.07^ij^	2.25 ± 0.03^ij^	2.25 ± 0.05^ij^	2.24 ± 0.07^ij^	2.25 ± 0.03^ij^	2.24 ± 0.09^ij^	2.20 ± 0.05^jk^
11	100 (−1)	100 (0)	90 (1)	20 (0)	2.57 ± 0.09^cd^	2.57 ± 0.02^cd^	2.57 ± 0.01^cd^	2.59 ± 0.02^c^	2.57 ± 0.07^cd^	2.57 ± 0.06^cd^	2.51 ± 004^de^
12	125 (0)	150 (1)	30 (−1)	20 (0)	2.27 ± 0.04^i^	2.27 ± 0.06^i^	2.27 ± 0.03^i^	2.27 ± 0.08^i^	2.27 ± 0.05^i^	2.26 ± 0.09^ij^	2.23 ± 0.07^ij^
13	100 (−1)	100 (0)	30 (−1)	20 (0)	2.56 ± 0.07^cd^	2.56 ± 0.04^cd^	2.56 ± 0.09^cd^	2.54 ± 0.03^d^	2.56 ± 0.06^cd^	2.52 ± 0.08^de^	2.37 ± 0.09^g^
14	150 (1)	50 (−1)	60 (0)	20 (0)	1.87 ± 0.03^o^	1.87 ± 0.09^o^	1.87 ± 0.05^o^	1.86 ± 0.04^o^	1.87 ± 0.07^o^	1.86 ± 0.05^o^	1.84 ± 0.03^op^
15	150 (1)	100 (0)	90 (1)	20 (0)	1.90 ± 0.09^no^	1.90 ± 0.08^no^	1.90 ± 0.04^no^	1.89 ± 0.07^no^	1.90 ± 0.03^no^	1.89 ± 0.05^no^	1.87 ± 0.01^o^
16	100 (−1)	100 (0)	60 (0)	30 (1)	2.74 ± 0.05^a^	2.74 ± 0.08^a^	2.74 ± 0.05^a^	2.74 ± 0.03^a^	2.74 ± 0.02^a^	2.73 ± 0.08^a^	2.64 ± 0.06^b^
17 (C3)	125 (0)	100 (0)	60 (0)	20 (0)	2.26 ± 0.08^ij^	2.26 ± 0.04^ij^	2.26 ± 0.03^ij^	2.26 ± 0.04^ij^	2.26 ± 0.01^ij^	2.25 ± 0.09^ij^	2.22 ± 0.04^j^
18	150 (1)	100 (0)	30 (−1)	20 (0)	1.89 ± 0.04^no^	1.89 ± 0.08^no^	1.89 ± 0.04^no^	1.89 ± 0.09^no^	1.89 ± 0.05^no^	1.88 ± 0.03^o^	1.85 ± 0.02^op^
19	100 (−1)	100 (0)	60 (0)	10 (−1)	2.46 ± 0.06^ef^	2.46 ± 0.09^ef^	2.46 ± 0.07^ef^	2.45 ± 0.03^ef^	2.44 ± 0.09^f^	2.40 ± 0.05^fg^	2.38 ± 0.07^g^
20	150 (1)	100 (0)	60 (0)	30 (1)	1.98 ± 0.04^m^	1.98 ± 0.03^m^	1.98 ± 0.01^m^	1.94 ± 0.08^mn^	1.98 ± 0.06^m^	1.94 ± 0.04^mn^	1.93 ± 0.08^n^
21(C4)	125 (0)	100 (0)	60 (0)	20 (0)	2.27 ± 0.05^i^	2.27 ± 0.07^i^	2.27 ± 0.07^i^	2.27 ± 0.04^i^	2.27 ± 0.07^i^	2.26 ± 0.05^ij^	2.24 ± 0.07^ij^
22	125 (0)	50 (−1)	60 (0)	10 (−1)	2.13 ± 0.08^kl^	2.13 ± 0.06^kl^	2.11 ± 0.04^l^	2.11 ± 0.06^l^	2.13 ± 0.09^kl^	2.11 ± 0.07^l^	2.09 ± 0.04^lm^
23 (C5)	125 (0)	100 (0)	60 (0)	20 (0)	2.27 ± 0.02^i^	2.27 ± 0.07^i^	2.27 ± 0.03^i^	2.27 ± 0.08^i^	2.27 ± 0.05^i^	2.26 ± 0.01^ij^	2.23 ± 0.07^ij^
24	100 (−1)	150 (1)	60 (0)	20 (0)	2.59 ± 0.09^c^	2.59 ± 0.03^c^	2.59 ± 0.07^c^	2.56 ± 0.03^cd^	2.59 ± 0.03^c^	2.57 ± 0.06^cd^	2.49 ± 0.08^e^
25	125 (0)	50 (−1)	60 (0)	30 (1)	2.35 ± 0.06^gh^	2.35 ± 0.04^gh^	2.35 ± 0.03^gh^	2.34 ± 0.07^gh^	2.35 ± 0.08^gh^	2.34 ± 0.05^gh^	2.32 ± 0.06^h^
26	125 (0)	100 (0)	30 (−1)	30 (1)	2.37 ± 0.08^g^	2.37 ± 0.02^g^	2.37 ± 0.08^g^	2.36 ± 0.03^gh^	2.37 ± 0.07^g^	2.36 ± 0.08^gh^	2.34 ± 0.09^gh^
27	125 (0)	150 (1)	90 (1)	20 (0)	2.28 ± 0.05^hi^	2.28 ± 0.07^hi^	2.28 ± 0.04^hi^	2.28 ± 0.02^hi^	2.28 ± 0.03^hi^	2.27 ± 0.09^i^	2.24 ± 0.07^ij^
28	125 (0)	150 (1)	60 (0)	30 (1)	2.38 ± 0.03^g^	2.38 ± 0.08^g^	2.38 ± 0.05^g^	2.38 ± 0.03^g^	2.38 ± 0.06^g^	2.37 ± 0.08^g^	2.35 ± 0.05^gh^
29	100 (−1)	50 (−1)	60 (0)	20 (0)	2.54 ± 0.01^d^	2.54 ± 0.04^d^	2.54 ± 0.03^d^	2.53 ± 0.08^de^	2.54 ± 0.05^d^	2.52 ± 0.07^de^	2.48 ± 0.02^ef^

Note

C1, C2, C3, C4, and C5 are the center points where extrusion processing was applied. Different superscripts letters indicate the significance difference (*p* ≤ .05) within the rows and columns within DHA.

Abbreviations: AA, arachidonic acid; BET, barrel exit temperature; FFR, feed flow rate; FMC, feed moisture content; FP, fish powder; SS, screw speed.

Moreover, retention for DHA in extrusion processing runs carried out at 150 BET, 10 FMC, and 30 FMC were significantly (*p* < .05) different from DHA retained in runs carried out at 150 BET with 20 FMC, whereas the SS and FFR did not significantly affect the retention of DHA in the final product. The DHA retention values of a run at 150 BET with 10 FMC and 30 FMC were significantly (*p* < .05) different from each other and also different from the runs with 20 FMC but runs with 20 FMC were nonsignificant (*p* > .05) from each other (Table 4). With 125 BET and 10 FMC, the value of runs was nonsignificant (*p* > .05) from each other but different from others at a similar temperature and different FMC. With 20 FMC, the values of runs were nonsignificant (*p* > .05) from each other but significantly (*p* < .05) different from the runs with 10 FMC and 30 FMC.

Similarly, the runs with 30 FMC having temperature were nonsignificant (*p* > .05) from each other but significantly (*p* < .05) different from others (Table 4). The values for the runs at 100 BET with 10 FMC and 30 FMC were significantly (*p* < .05) different from each other and also from the runs with 20 FMC, but the runs with 20 FMC had nonsignificant (*p* > .05) values from each other but these runs were significantly (*p* < .05) different from the runs with 10 FMC and 30 FMC. The DHA retention was highest in the run with 100 BET and 30 FMC as displayed in Table 4. Similar to DHA, the retention values of EPA in the runs with 20 FMC and 150 BET were nonsignificant (*p* > .05) from each other but significantly (*p* > .05) different from the runs with 10 FMC and 30 FMC (Table 5). The results for runs with 10 FMC and 30 FMC with a similar temperature for 150 BET were significantly different from each other and also different from the runs with 20 FMC. The retention value of EPA in the runs with 125 BET and 20 FMC was nonsignificant (*p* > .05) between each other but significantly (*p* > .05) different from the runs with FMC of 10 and 30. The run values with 10 FMC were also nonsignificant from each other but different from the rest two.

Similarly, the EPA retention in the runs with 30 FMC was nonsignificant (*p* > .05) from each other but significantly (*p* > .05) different from the EPA values in the runs with 10 and 20 FMC. At 100 BET the values of the runs with 10, 20, and 30 FMC were significantly (*p* > .05) different from each other but the values in the runs with 20 FMC were nonsignificant (*p* > .05) from each other as depicted in Table 5. The retention of the AA was also observed in three different temperatures at 150 BET, 125 BET, and 100 BET. The values of the runs at 150 with 30, 10, and 20 FMC were significantly (*p* > .05) different from each other. The AA retention in the runs with 20 FMC with a similar temperature of 150 BET was nonsignificant (*p* > .05) from each other. When the BET was set at 125 BET the AA values in the runs with 10, 20, and 30 FMC were significantly (*p* > .05) different from each other but the values of runs with 20 FMC were nonsignificant from each other and this condition was similar for the runs with 10 FMC and 30 FMC. With 10 FMC and 30 FMC, the values of AA in the runs were different from each other and the values of the runs with 20 FMC were nonsignificant (*p* > .05) from each other and significantly (*p* > .05) different from the runs with 10 FMC and 30 FMC (Table 6).

### 3.3 Storage stability of DHA, EPA, and AA

Storage stability of DHA, EPA, and AA was observed from 0 days to 1 month, 3 months, and 6 months at different processing conditions (with storage temperatures of 4°C and 25°C) (Tables 4, 5, 6). For DHA, there was nonsignificant (*p* > .05) change observed in 1 month and 3 months at 4°C and 25°C, with different processing conditions, but on 6 months storage time, the significantly (*p* > .05) different values were observed at 25°C in different runs (5, 11, 13, 16, 19, 24, 25, 26, 28 and 29). Under extrusion condition of 100 BET with 10, 20, and 30 FMC, a significant (*p* > .05) change was determined for DHA. Also, significant (*p* > .05) change for DHA was recorded for 125 BET with 30 FMC, at 25°C storage temperature, but nonsignificant (*p* > .05) results at 4°C for 6 months of storage was determined. Nonsignificant (*p* > .05) changes observed in other processing conditions are presented in Table 4.

Furthermore, for EPA, significantly (*p* > .05) different estimations were determined for 6 months of storage at 25°C with extrusion conditions of 100 BET using 10, 20, and 30 FMC in 11, 13, 16, 19, 24, and 29 runs and with 5, 25, 26, and 28 runs.

Likewise, significant (*p* > .05) changes were also observed with extrusion conditions of 125 BET with 30 FMC. While nonsignificant (*p* > .05) changes were determined on 1 and 3 months at 4°C and 25°C storage temperatures, on 6 months of storage at 4°C temperature, nonsignificant (*p* > .05) changes were recorded (Table 5).

Additionally, for AA, nonsignificant (*p* > .05) changes were determined during 1, 3, and 6 (at 4°C) months of storage but a significant (*p* > .05) change in the storage stability for AA was determined during 6 months at 25°C temperature. All the runs (11, 13, 16, 19, 24, and 29) delivered a significant change in AA value. The extrusion conditions for these runs were 100 BET with 10, 20, and 30 FMC. The values in runs with 100 BET with 20 FMC were significantly (*p* > .05) different from the 10 FMC and 30 FMC results. The runs with 10 FMC and 30 FMC were also different from each other as depicted in Table 6. The runs with 100 BET and 30 FMC had greater AA values than the others. Therefore, it can be concluded that two factors (BET and FMC) affect the storage stability and retention of DHA, EPA, and AA in extrusion conditions.

### 3.4 Oxidative stability of EFP

Maximum PV was noted as 1.72 ± 0.04 (at 0 days) with 150 BET and 10 FMC, while the minimum estimation was 1.08 ± 0.01 at 100 BET and 30 FMC. Significantly different outcomes for PV were determined at 4°C and 25°C with different processing conditions (Table 7). Also, nonsignificant findings were recorded at 4°C and 25°C using 1, 14, 15, and 18 runs with 150 BET and 20 FMC while at 150 BET (with 10 and 30 FMC) results were significantly (*p* > .05) changed from the others.

**TABLE 7:** Changes in PV in EFP at different temperature and storage intervals

Extrusion processing run	Independent variables				Dependent variable PV (meq/kg fat)						
	Barrel exit temp. (BET) °C	Screw speed (SS)	Feed flow rate (FFR)	Feed moisture content (FMC)							
					0 day	Storage stability at 4°C			Storage stability at 25°C		
						1 month	3 months	6 months	1 month	3 months	6 months
1	150 (1)	150 (1)	60 (0)	20 (0)	1.60 ± 0.08^j^	1.85 ± 0.04^ij^	3.21 ± 0.08^ghi^	7.54 ± 0.02^bcd^	3.97 ± 0.02^g^	6.56 ± 0.02^cde^	9.90 ± 0.05^a^
2 (C1)	125 (0)	100 (0)	60 (0)	20 (0)	1.38 ± 0.01^jk^	1.53 ± 0.03^ijk^	2.79 ± 0.07^hi^	6.69 ± 0.01^d^	3.39 ± 0.08^h^	5.65 ± 0.06^def^	8.79 ± 0.03^abc^
3	125 (0)	150 (1)	60 (0)	10 (−1)	1.46 ± 0.04^ijk^	1.62 ± 0.02^j^	2.98 ± 0.09^hi^	6.82 ± 0.04^d^	3.61 ± 0.02^gh^	5.98 ± 0.02^e^	9.37 ± 0.03^b^
4	125 (0)	100 (0)	30 (−1)	10 (−1)	1.45 ± 0.09^ijk^	1.63 ± 0.03^j^	3.01 ± 0.02^hi^	6.83 ± 0.02^d^	3.59 ± 0.05^gh^	5.99 ± 0.09^e^	9.36 ± 0.02^b^
5	125 (0)	100 (0)	90 (1)	30 (1)	1.29 ± 0.07^jk^	1.40 ± 0.08^jk^	3.51 ± 0.03^h^	6.56 ± 0.03^cde^	3.15 ± 0.03^ghi^	5.28 ± 0.08^ef^	8.19 ± 0.05^bc^
6	150 (1)	100 (0)	60 (0)	10 (−1)	1.72 ± 0.04^j^	1.91 ± 0.02^ij^	3.46 ± 0.02^h^	7.81 ± 0.08^c^	4.21 ± 0.04^fg^	7.05 ± 0.03^cd^	9.98 ± 0.03^a^
7	125 (0)	100 (0)	90 (1)	10 (−1)	1.46 ± 0.02^ijk^	1.63 ± 0.08^j^	2.99 ± 0.06^hi^	6.84 ± 0.08^d^	3.63 ± 0.02^gh^	5.98 ± 0.02^e^	9.35 ± 0.03^b^
8 (C2)	125 (0)	100 (0)	60 (0)	20 (0)	1.39 ± 0.04^jk^	1.54 ± 0.06^ijk^	2.80 ± 0.07^hi^	6.70 ± 0.03^d^	3.40 ± 0.03^h^	5.64 ± 0.02^def^	8.80 ± 0.01^abc^
9	125 (0)	50 (−1)	90 (1)	20 (0)	1.39 ± 0.06^jk^	1.53 ± 0.06^ijk^	2.81 ± 0.09^hi^	6.70 ± 0.03^d^	3.41 ± 0.03^h^	5.69 ± 0.08^def^	8.83 ± 0.01^abc^
10	125 (0)	50 (−1)	30 (−1)	20 (0)	1.40 ± 0.02^jk^	1.55 ± 0.04^ijk^	2.84 ± 0.06^hi^	6.72 ± 0.08^d^	3.40 ± 0.08^h^	5.68 ± 0.08^def^	8.84 ± 0.04^abc^
11	100 (−1)	100 (0)	90 (1)	20 (0)	1.13 ± 0.08^k^	1.21 ± 0.02^k^	3.23 ± 0.02^ghi^	6.26 ± 0.05^de^	2.75 ± 0.02^i^	4.71 ± 0.03^f^	7.03 ± 0.02^cd^
12	125 (0)	150 (1)	30 (−1)	20 (0)	1.13 ± 0.04^k^	1.53 ± 0.06^ijk^	2.83 ± 0.09^hi^	6.67 ± 0.06^d^	2.39 ± 0.01^hij^	4.67 ± 0.06^f^	8.80 ± 0.05^abc^
13	100 (−1)	100 (0)	30 (−1)	20 (0)	1.09 ± 0.01^k^	1.23 ± 0.02^k^	3.12 ± 0.04^ghi^	6.28 ± 0.09^de^	2.70 ± 0.03^i^	4.46 ± 0.08^efg^	7.02 ± 0.08^cd^
14	150 (1)	50 (−1)	60 (0)	20 (0)	1.65 ± 0.07^j^	1.83 ± 0.05^ij^	3.18 ± 0.04^ghi^	7.57 ± 0.02^bcd^	4.01 ± 0.03^g^	6.59 ± 0.03^cde^	9.91 ± 0.06^a^
15	150 (1)	100 (0)	90 (1)	20 (0)	1.62 ± 0.03^j^	1.84 ± 0.06^ij^	3.20 ± 0.02^ghi^	7.56 ± 0.06^bcd^	4.00 ± 0.01^g^	6.64 ± 0.04^cde^	9.90 ± 0.06^a^
16	100 (−1)	100 (0)	60 (0)	30 (1)	1.08 ± 0.01^k^	1.86 ± 0.03^ij^	2.79 ± 0.02^hi^	6.18 ± 0.09^de^	2.66 ± 0.04^i^	4.42 ± 0.06^efg^	6.86 ± 0.05^d^
17 (C3)	125 (0)	100 (0)	60 (0)	20 (0)	1.37 ± 0.05^jk^	1.53 ± 0.05^ijk^	2.78 ± 0.07^hi^	6.69 ± 0.05^d^	3.39 ± 0.02^h^	5.66 ± 0.04^def^	8.79 ± 0.05^abc^
18	150 (1)	100 (0)	30 (−1)	20 (0)	1.64 ± 0.05^j^	1.83 ± 0.02^ij^	3.19 ± 0.04^ghi^	7.55 ± 0.01^bcd^	4.01 ± 0.05^g^	6.60 ± 0.08^cde^	9.91 ± 0.01^a^
19	100 (−1)	100 (0)	60 (0)	10 (−1)	1.17 ± 0.03^k^	1.30 ± 0.04^jk^	3.04 ± 0.08^hi^	6.36 ± 0.08^de^	2.88 ± 0.05^hi^	4.79 ± 0.04^f^	7.43 ± 0.03^bcd^
20	150 (1)	100 (0)	60 (0)	30 (1)	1.54 ± 0.02^ijk^	1.75 ± 0.01^j^	3.12 ± 0.06^ghi^	7.15 ± 0.05^cd^	3.81 ± 0.03^fgh^	6.31 ± 0.08^de^	9.78 ± 0.07^ab^
21(C4)	125 (0)	100 (0)	60 (0)	20 (0)	1.38 ± 0.02^jk^	1.55 ± 0.08^ijk^	2.81 ± 0.07^hi^	6.68 ± 0.07^d^	3.40 ± 0.04^h^	5.65 ± 0.06^def^	8.78 ± 0.08^abc^
22	125 (0)	50 (−1)	60 (0)	10 (−1)	1.48 ± 0.08^ijk^	1.64 ± 0.04^j^	3.03 ± 0.08^hi^	6.86 ± 0.04^d^	3.61 ± 0.04^gh^	6.03 ± 0.06^e^	9.40 ± 0.09^b^
23 (C5)	125 (0)	100 (0)	60 (0)	20 (0)	1.39 ± 0.07^jk^	1.54 ± 0.04^ijk^	2.80 ± 0.07^hi^	6.70 ± 0.03^d^	3.41 ± 0.01^h^	5.66 ± 0.08^def^	8.80 ± 0.01^abc^
24	100 (−1)	150 (1)	60 (0)	20 (0)	1.15 ± 0.02^k^	1.22 ± 0.01^k^	3.19 ± 0.06^ghi^	6.27 ± 0.08^de^	2.74 ± 0.02^i^	4.71 ± 0.03^f^	7.03 ± 0.07^cd^
25	125 (0)	50 (−1)	60 (0)	30 (1)	1.30 ± 0.05^jk^	1.42 ± 0.06^ijk^	3.37 ± 0.09^h^	6.60 ± 0.02^cde^	3.13 ± 0.01^ghi^	5.32 ± 0.06^ef^	8.22 ± 0.03^b^c
026	125 (0)	100 (0)	30 (−1)	30 (1)	1.31 ± 0.01^jk^	1.39 ± 0.06^jk^	3.33 ± 0.02^h^	6.57 ± 0.02^cde^	3.17 ± 0.04^ghi^	5.33 ± 0.02^ef^	8.23 ± 0.06^bc^
27	125 (0)	150 (1)	90 (1)	20 (0)	1.37 ± 0.06^jk^	1.50 ± 0.04^ijk^	3.36 ± 0.08^h^	6.67 ± 0.06^d^	3.38 ± 0.09^h^	5.65 ± 0.04^def^	8.79 ± 0.05^abc^
28	125 (0)	150 (1)	60 (0)	30 (1)	1.28 ± 0.08^jk^	1.38 ± 0.04^jk^	3.48 ± 0.08^h^	6.58 ± 0.02^cde^	3.16 ± 0.02^ghi^	5.29 ± 0.06^ef^	8.20 ± 0.07^bc^
29	100 (−1)	50 (−1)	60 (0)	20 (0)	1.10 ± 0.02^k^	1.24 ± 0.02^k^	3.13 ± 0.04^ghi^	6.28 ± 0.09^de^	2.71 ± 0.03^i^	4.51 ± 0.02^efg^	6.99 ± 0.01^cd^

Note

C1, C2, C3, C4, and C5 are the center points where extrusion processing was applied. Different superscripts letters indicate the significance difference (*p* ≤ .05) within the rows and columns within DHA.

Abbreviations: BET, barrel exit temperature; FFR, feed flow rate; FMC, feed moisture content; FP, fish powder; PV, peroxide value; SS, screw speed.

Similarly, the values in the runs with 125 BET and 20 FMC at 4°C and 25°C were nonsignificantly different but in the runs with BET 125 and 10 FMC significantly (*p* > .05) changed from the others whereas nonsignificantly changed from each other. Runs with 30 FMC with 125 BET were also nonsignificantly different from each other but significantly (*p* > .05) different from the others with dissimilar FMC and BET. The values in the runs with 100 BET with 10 FMC and 30 FMC were significantly (*p* > .05) changed from each other and also from the others. Likewise, the values for 100 BET and 20 FMC were nonsignificantly changed from each other but significantly (*p* > .05) different from the others (Table 7).

The maximum value observed for TBARS at 0 days with 150 BET and 10 FMC was 0.135 ± 0.008 and the minimum value examined at 100 BET and 30 FMC was 0.006 ± 0.001 as shown in Table 8. The values for TBARS at 4°C and 25°C were significantly different in 1, 3, and 6 months at different processing conditions (Table 8).

**TABLE 8:** TBARS value at different temperature and storage intervals

Extrusion processing run	Independent variables				Dependent variable TBARS (mg MDA/kg fat)						
	Barrel exit temp. (BET) °C	Screw speed (SS)	Feed flow rate (FFR)	Feed moisture content (FMC)							
					0 day	Storage stability at 4°C			Storage stability at 25°C		
						1 month	3 months	6 months	1 month	3 months	6 months
1	150 (1)	150 (1)	60 (0)	20 (0)	0.115 ± 0.003^hi^	0.127 ± 0.003^h^	0.160 ± 0.004^gh^	0.204 ± 0.002^fg^	0.140 ± 0.005^gh^	0.301 ± 0.004^e^	0.788 ± 0.002^b^
2 (C1)	125 (0)	100 (0)	60 (0)	20 (0)	0.041 ± 0.006^ij^	0.051 ± 0.004^ij^	0.075 ± 0.005^i^	0.110 ± 0.006^hi^	0.071 ± 0.008^i^	0.129 ± 0.009^h^	0.331 ± 0.006^e^
3	125 (0)	150 (1)	60 (0)	10 (−1)	0.066 ± 0.001^ij^	0.077 ± 0.003^i^	0.095 ± 0.006^hi^	0.128 ± 0.003^h^	0.094 ± 0.006^hi^	0.223 ± 0.005^f^	0.570 ± 0.002^d^
4	125 (0)	100 (0)	30 (−1)	10 (−1)	0.069 ± 0.002^ij^	0.078 ± 0.001^i^	0.097 ± 0.007^hi^	0.132 ± 0.007^gh^	0.100 ± 0.002^hi^	0.227 ± 0.007^f^	0.579 ± 0.009^d^
5	125 (0)	100 (0)	90 (1)	30 (1)	0.023 ± 0.005^j^	0.033 ± 0.006^ij^	0.047 ± 0.004^ij^	0.074 ± 0.003^i^	0.047 ± 0.001^ij^	0.062 ± 0.001^ij^	0.316 ± 0.007^e^
6	150 (1)	100 (0)	60 (0)	10 (−1)	0.135 ± 0.008^gh^	0.143 ± 0.008^gh^	0.177 ± 0.002^g^	0.232 ± 0.009^f^	0.158 ± 0.003^gh^	0.338 ± 0.002^e^	0.863 ± 0.006^a^
7	125 (0)	100 (0)	90 (1)	10 (−1)	0.067 ± 0.004^ij^	0.078 ± 0.009^i^	0.096 ± 0.006^hi^	0.130 ± 0.003^gh^	0.098 ± 0.006^hi^	0.225 ± 0.008^f^	0.574 ± 0.002^d^
8 (C2)	125 (0)	100 (0)	60 (0)	20 (0)	0.042 ± 0.009^ij^	0.052 ± 0.009^ij^	0.074 ± 0.003^i^	0.111 ± 0.002^hi^	0.072 ± 0.008^i^	0.128 ± 0.009^h^	0.332 ± 0.006^e^
9	125 (0)	50 (−1)	90 (1)	20 (0)	0.043 ± 0.002^ij^	0.053 ± 0.002^ij^	0.073 ± 0.004^i^	0.113 ± 0.005^hi^	0.074 ± 0.003^i^	0.130 ± 0.007^gh^	0.335 ± 0.003^e^
10	125 (0)	50 (−1)	30 (−1)	20 (0)	0.044 ± 0.007^ij^	0.056 ± 0.002^ij^	0.077 ± 0.004^i^	0.115 ± 0.001^hi^	0.075 ± 0.009^i^	0.130 ± 0.003^gh^	0.336 ± 0.009^e^
11	100 (−1)	100 (0)	90 (1)	20 (0)	0.012 ± 0.003^j^	0.018 ± 0.001^j^	0.029 ± 0.009^j^	0.046 ± 0.003^ij^	0.018 ± 0.005^j^	0.035 ± 0.007^ij^	0.093 ± 0.006^hi^
12	125 (0)	150 (1)	30 (−1)	20 (0)	0.040 ± 0.008^ij^	0.051 ± 0.009^ij^	0.072 ± 0.003^i^	0.109 ± 0.002^hi^	0.070 ± 0.008^i^	0.126 ± 0.009^h^	0.229 ± 0.006^f^
13	100 (−1)	100 (0)	30 (−1)	20 (0)	0.014 ± 0.006^j^	0.019 ± 0.009^j^	0.029 ± 0.007^j^	0.047 ± 0.009^ij^	0.018 ± 0.005^j^	0.038 ± 0.008^ij^	0.097 ± 0.003^hi^
14	150 (1)	50 (−1)	60 (0)	20 (0)	0.120 ± 0.009^h^	0.130 ± 0.008^gh^	0.164 ± 0.001^gh^	0.214 ± 0.001^fg^	0.144 ± 0.006^gh^	0.312 ± 0.001^e^	0.796 ± 0.004^b^
15	150 (1)	100 (0)	90 (1)	20 (0)	0.116 ± 0.002^hi^	0.129 ± 0.004^h^	0.161 ± 0.008^gh^	0.210 ± 0.006^fg^	0.146 ± 0.007^gh^	0.308 ± 0.003^e^	0.793 ± 0.004^b^
16	100 (−1)	100 (0)	60 (0)	30 (1)	0.006 ± 0.001^j^	0.011 ± 0.001^j^	0.015 ± 0.003^j^	0.021 ± 0.001^j^	0.016 ± 0.005^j^	0.036 ± 0.001^ij^	0.096 ± 0.003^hi^
17 (C3)	125 (0)	100 (0)	60 (0)	20 (0)	0.044 ± 0.003^ij^	0.051 ± 0.007^ij^	0.073 ± 0.008^i^	0.109 ± 0.002^hi^	0.073 ± 0.008^i^	0.128 ± 0.009^h^	0.331 ± 0.006^e^
18	150 (1)	100 (0)	30 (−1)	20 (0)	0.119 ± 0.005^hi^	0.129 ± 0.002^h^	0.163 ± 0.004^gh^	0.208 ± 0.004^fg^	0.141 ± 0.001^gh^	0.310 ± 0.004^e^	0.790 ± 0.002^b^
19	100 (−1)	100 (0)	60 (0)	10 (−1)	0.015 ± 0.007^j^	0.024 ± 0.007^j^	0.038 ± 0.001^ij^	0.052 ± 0.002^ij^	0.021 ± 0.005^j^	0.044 ± 0.008^ij^	0.107 ± 0.006^hi^
20	150 (1)	100 (0)	60 (0)	30 (1)	0.109 ± 0.004^hi^	0.121 ± 0.001^h^	0.148 ± 0.006^gh^	0.170 ± 0.006^g^	0.128 ± 0.004^h^	0.234 ± 0.006^f^	0.697 ± 0.008^c^
21(C4)	125 (0)	100 (0)	60 (0)	20 (0)	0.043 ± 0.006^ij^	0.051 ± 0.001^ij^	0.074 ± 0.001^i^	0.111 ± 0.002^hi^	0.071 ± 0.008^i^	0.127 ± 0.009^h^	0.330 ± 0.006^e^
22	125 (0)	50 (−1)	60 (0)	10 (−1)	0.070 ± 0.008^i^	0.079 ± 0.001^i^	0.100 ± 0.007^hi^	0.135 ± 0.001^gh^	0.103 ± 0.002^hi^	0.228 ± 0.007^f^	0.589 ± 0.009^d^
23 (C5)	125 (0)	100 (0)	60 (0)	20 (0)	0.041 ± 0.003^ij^	0.053 ± 0.009^ij^	0.075 ± 0.009^i^	0.110 ± 0.002^hi^	0.073 ± 0.008^i^	0.128 ± 0.009^h^	0.332 ± 0.006^e^
24	100 (−1)	150 (1)	60 (0)	20 (0)	0.013 ± 0.001^j^	0.017 ± 0.003^j^	0.027 ± 0.001^j^	0.051 ± 0.004^ij^	0.018 ± 0.005^j^	0.031 ± 0.006^ij^	0.107 ± 0.003^hi^
25	125 (0)	50 (−1)	60 (0)	30 (1)	0.026 ± 0.007^j^	0.036 ± 0.002^ij^	0.052 ± 0.002^ij^	0.081 ± 0.002^hi^	0.050 ± 0.002^ij^	0.065 ± 0.009^ij^	0.318 ± 0.007^e^
026	125 (0)	100 (0)	30 (−1)	30 (1)	0.024 ± 0.004^j^	0.034 ± 0.008^ij^	0.049 ± 0.004^ij^	0.077 ± 0.004^i^	0.049 ± 0.002^ij^	0.064 ± 0.009^ij^	0.316 ± 0.002^e^
27	125 (0)	150 (1)	90 (1)	20 (0)	0.039 ± 0.003^ij^	0.050 ± 0.003^ij^	0.070 ± 0.001^i^	0.105 ± 0.008^hi^	0.069 ± 0.006^ij^	0.124 ± 0.002^h^	0.321 ± 0.006^e^
28	125 (0)	150 (1)	60 (0)	30 (1)	0.021 ± 0.006^j^	0.032 ± 0.003^ij^	0.043 ± 0.002^ij^	0.071 ± 0.009^i^	0.045 ± 0.002^ij^	0.067 ± 0.009^ij^	0.315 ± 0.007^e^
29	100 (−1)	50 (−1)	60 (0)	20 (0)	0.014 ± 0.001^j^	0.016 ± 0.009^j^	0.030 ± 0.007^ij^	0.049 ± 0.004^ij^	0.019 ± 0.005^j^	0.040 ± 0.008^ij^	0.097 ± 0.006^hi^

Note

C1, C2, C3, C4, and C5 are the center points where extrusion processing was applied. Different superscripts letters indicate the significance difference (*p* ≤ .05) within the rows and columns within DHA.

Abbreviations: BET, barrel exit temperature; FFR, feed flow rate; FMC, feed moisture content; FP, fish powder; SS, screw speed; TBARS, thiobarbituric acid reactive substances values.

## 4 DISCUSSION

In addition to other health benefits, fish and fish products are vital for cardiac health and the proper functions of the eye. Fish is a good source of PUFAs (DHA, AA, and EPA) which have gained consumer attention in the last three decades. Therefore, we processed fish into powder which was a colorless and odor product. Protein content dominates (75%) in the product with essential minerals and fat content; therefore, the EFP can be incorporated in food items as a source of protein, minerals, and fat. One type of fish differs from others due to the presence of long‐chain PUFAs, DHA, or configuration of the fatty acids profile.

However, the design of the dryer, liquid material, operating conditions, and fish composition are responsible for the end product and dried powder (Deis, 1997). Keeping in view the importance of PUFAs for human health, we also studied their storage stability at two different temperatures (4°C and 25°C) for 1, 3, and 6 months with different processing conditions.

Some recent studies were also reported on the nutritional significance of fish and fish products. For instance, Abbey et al. (2017) prepared FP from different species and determined 28 to 80 g/100 g crude proteins, 4.7 to 11.5/100 g crude fat, 3.4 to 14.2 g ash content, and 3.5 to 8.4 (g/100 g) moisture content, which were comparable with our findings. Also, carbohydrate content ranged from 5.39 to 7.53 g/100 g, which was not comparable to our findings. The difference in results might be due to different processing conditions and types of fish used.

In another study, Pansawat et al. (2004) stated that the retention of EPA in fish, rice‐based snacks was maximum at extreme conditions of SS and FMC that are by the present study.

Whereas Kostadinović et al. (2016) stated that extrusion treatment increases the amounts of DHA, we observed that with the increase in FMC, DHA also increases as BET decreases. Moreover, Björck and Asp (1983) revealed that the higher extrusion temperature can decrease the fat content in the final product and this conclusion can be comparable to current findings. This might be due to the expulsion of fumes at a higher temperature from fats. So, the change in parameters such as BET, SS, and FMC also affects the number of fats on different levels. BET and FMC variables affect the composition retention and storage stability of PUFAs.

Additionally, Fitzpatrick et al. (2004) also stated that storage conditions may influence the levels of fat and the same trend was also noticed in our study. With the change in BET, SS and FMC also change the levels of fat. Additionally, Lohani and Muthukumarappan (2017) evaluated extruded products and stated that extrusion procedures help to increase the antioxidant activities and make the stable oxidation.

## 5 CONCLUSION

Nowadays, the dried fish powder (FP) is extensively applied for the production of value‐added foods for improvement in functional and nutritional value. The current study was designed to optimize the process for ready‐to‐eat extruded fish powder preparation, and then, its bioactive fatty acid profile was characterized. The Rohu fish meat was minced, extruded, and microwave vacuum‐dried. Our results established that thermal extrusion and microwave vacuum drying have the potential to improve the bioactive fatty acids, PV, and TBARS of FP. The findings show the importance of the proposed method for the food industry to prepare nutritious value‐added functional products.

## CONFLICT OF INTEREST

The authors declare have no conflict of interest to declare.

## Data Availability

Dataset support the conclusion, included with in the article.
